# Hierarchical clustering of gene expression patterns in the Eomes + lineage of excitatory neurons during early neocortical development

**DOI:** 10.1186/1471-2202-13-90

**Published:** 2012-08-01

**Authors:** David A Cameron, Frank A Middleton, Anjen Chenn, Eric C Olson

**Affiliations:** 1 Department of Neuroscience and Physiology, SUNY Upstate Medical University, Syracuse, NY 13210, USA; 2 Department of Electrical Engineering and Computer Science, Syracuse University, Syracuse, NY 13210, USA; 3 Department of Pathology, Feinberg School of Medicine, Northwestern University, Chicago IL, 60611, USA; 4 Department of Neuroscience and Physiology, SUNY Upstate Medical University, 3295 Weiskotten Hall, 750 E, Adams St. Syracuse, NY 13210, USA

**Keywords:** Transcriptome, Migration, Cortex, Profiling, Excitatory neuron

## Abstract

**Background:**

Cortical neurons display dynamic patterns of gene expression during the coincident processes of differentiation and migration through the developing cerebrum. To identify genes selectively expressed by the Eomes + (Tbr2) lineage of excitatory cortical neurons, GFP-expressing cells from Tg(Eomes::eGFP) Gsat embryos were isolated to > 99% purity and profiled.

**Results:**

We report the identification, validation and spatial grouping of genes selectively expressed within the Eomes + cortical excitatory neuron lineage during early cortical development. In these neurons 475 genes were expressed ≥ 3-fold, and 534 genes ≤ 3-fold, compared to the reference population of neuronal precursors. Of the up-regulated genes, 328 were represented at the Genepaint in situ hybridization database and 317 (97%) were validated as having spatial expression patterns consistent with the lineage of differentiating excitatory neurons. A novel approach for quantifying in situ hybridization patterns (QISP) across the cerebral wall was developed that allowed the hierarchical clustering of genes into putative co-regulated groups. Forty four candidate genes were identified that show spatial expression with Intermediate Precursor Cells, 49 candidate genes show spatial expression with Multipolar Neurons, while the remaining 224 genes achieved peak expression in the developing cortical plate.

**Conclusions:**

This analysis of differentiating excitatory neurons revealed the expression patterns of 37 transcription factors, many chemotropic signaling molecules (including the Semaphorin, Netrin and Slit signaling pathways), and unexpected evidence for non-canonical neurotransmitter signaling and changes in mechanisms of glucose metabolism. Over half of the 317 identified genes are associated with neuronal disease making these findings a valuable resource for studies of neurological development and disease.

## Background

Excitatory, or projection, neurons comprise ~85% of the neuronal population in the mature cerebral cortex. These neurons share a stereotypical developmental trajectory, with most generated in the neocortical ventricular zone (VZ) and subsequently migrating outward, through complex and changing micro-environments, towards the cortical surface. During this radial migration neurons adopt multiple morphologies and display several distinct modes of migration [1].

The period of radial migration and early differentiation coincides with the sequential expression of a network of transcription factors. Core elements of the network include Pax6, Neurogenin, Neurod, Eomes (Tbr2), and Tbr1 [2-4]. Some of the network members are known to be essential for proper migration [5,6], axogenesis [7,8], dendritogenesis [9], and adoption of neuronal subtype [10-12]. The ability of neurons to simultaneously navigate through the developing cortex and acquire characteristic phenotypes is thus likely to depend on dynamic patterns of gene expression during the early postmitotic period.

Characterization of these cell-intrinsic, dynamic gene expression patterns remains incomplete. Transcriptional profiling of microdissected samples derived from developing brain, although revealing regional and temporal differences across the brain [13-15] is limited by the inclusion of blood vessel endothelial cells, inhibitory neurons, and glia within the dissected tissue: the derived transcriptional profile necessarily reflects contributions of multiple, and often non-neuronal, cell types. These interpretive obstacles can in principle be eliminated with a combined application of cell sorting from acutely dissociated brain [16] and subsequent transcriptional profiling [17,18].

In this study fluorescence activated cell (FAC) sorting was used to purify neurons of the excitatory cortical lineage, from which RNA was isolated for transcriptional profiling. We focused on the neuronal lineage that expresses Eomes (Tbr2), a transcription factor that is expressed by intermediate precursor cells [19] and has been genetically linked to microcephaly in mouse [20] and human [21]. The target neurons were acutely dissociated at E14.5 from a reporter transgenic mouse (Eomes::eGFP)Gsat in which immature neurons of the excitatory lineage express eGFP [22]. The Genepaint in situ database [23,24], which provides comprehensive coverage of gene expression patterns in mouse embryos at E14.5 was then utilized to validate the transcriptional profiling, from which dynamically expressed genes were identified. A simple but robust technique was developed to quantify the optical density of the in situ patterns (QISPs) across the cerebral wall. These patterns were in turn hierarchically clustered into putative functional groups.

## Methods

### Mice

All animal procedures were approved by the IACUC of SUNY Upstate Medical University. Tg(Eomes::eGFP)Gsat mice (The Gene Expression Nervous System Atlas Project, NINDS Contract # N01NS02331 to The Rockefeller University, New York, NY) were mated to C57Bl6 (Taconic Labs, Germantown, NY) to generate embryos. The day of plug discovery was designated embryonic day 0.5 (E0.5). Eomesodermin (Eomes) – also known as Tbr2 – is a transcription factor of the T-box family expressed by intermediate precursor cells in the excitatory neuron lineage of the cortex [3,25]. Eomes::eGFP mice contain a BAC transgene with 185 kb of 5’ and 18 kb of 3’ sequence surrounding the Eomes locus. In these mice selective GFP expression is observed in developing excitatory neurons indicating the fidelity of the large *cis*-regulatory elements in the BAC transgene [26].

### Tissue dissociation and cell sorting

**Figure 1:**
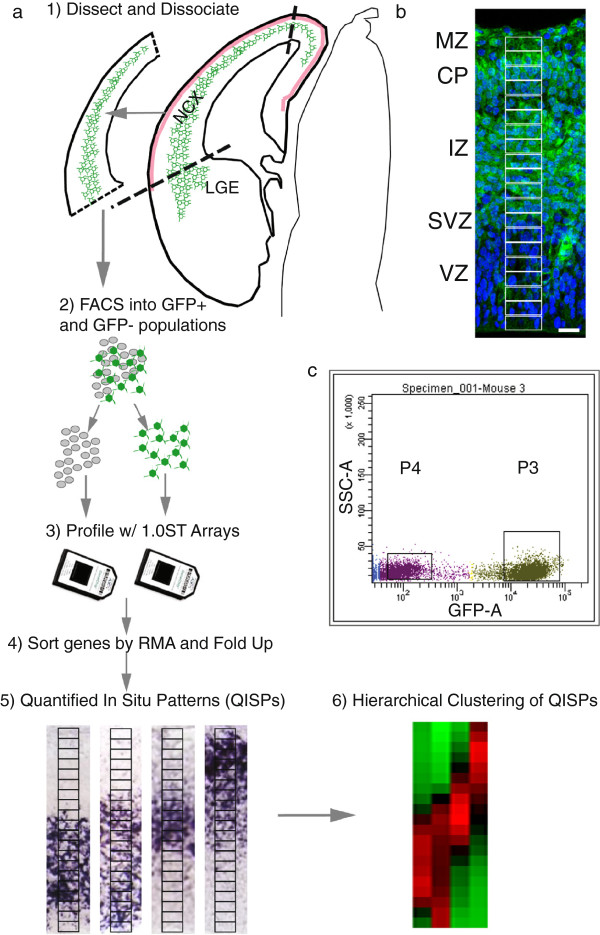
**Isolation, profiling, and QISP analysis of GFP + (Eomes+) cortical neurons. *a***, Neocortex (NCX) containing GFP + neurons is dissected, and cells isolated with flourescence activated cell sorting (FACS). Following transcriptional profiling, genes are sorted by RMA and their level of fold up- or down-regulation compared to the FACS GFP- population. Verification of up-regulated genes is accomplished with quantified in situ hybridization (QISP), and the verified genes hierarchically clustered (see Methods). ***b***, Fluorescence microscopic images of a section from an E14.5 Eomes::eGFP brain, displaying GFP + (green) and GFP- cells, that are blue due to Hoechst nuclear labeling. Subregions of Interest (sROIs) 1-20 are superimposed in white on the image. Scale bar, 25 μm. MZ, marginal zone; CP, cortical plate; IZ, intermediate zone; SVZ, subventricular zone; VZ, ventricular zone; LGE, lateral ganglionic eminence. ***c***, Representative outcome of FAC sort of GFP + and GFP- cells. A GFP + (P3) and GFP- (P4) were isolated based on fluorescence (GFP-A) and side scatter (SSC-A). The boxes denote the relevant sorted populations. All in situ hybridizations were obtained from Genepaint.

To generate RNA from enhanced Green Fluorescent Protein (eGFP) expressing neurons, dorsal neocortices from Tg(Eomes::eGFP)Gsat embryos were rapidly dissected at E13.5 and E14.5. The meninges were removed and the cortical tissue was dissociated in 0.25% Trypsin-EDTA solution (Invitrogen) for subsequent fluorescence activated cell (FAC) sorting (Figure 1). Cortices from 2 embryos were used per sorted sample and 3 samples were generated per developmental time point. Dissociated cells were kept in ice-cold buffer (PBS + 2% BSA) until FAC sorting, with the total elapsed time between dissection and sorting < 1 hr. Cell suspensions were sorted into GFP + and GFP- populations on a Becton Dickinson FACS Vantage Flow Cytometer Cell Sorter (SUNY Upstate Medical Flow Cytometry Unit). Sorting continued until a minimum of 100,000 GFP + cells were generated per sample. The total time required for sorting the 3 samples was less than 30 min. Cells were sorted directly into collection tubes containing RNALater™(Qiagen Inc., Valencia, CA) to minimize post-sort RNA degradation.

### RNA isolation and quality assessment

High quality total RNA was isolated using the RNAeasy™ (Qiagen) kit and flash frozen. From a total volume of 35 μl, 1 μl of the RNAeasy fluid was run on an Agilent 2100 Bioanalyzer using the RNA PicoChip to assess RNA structural quality and quantity. Peak areas for 28 S and 18 S rRNA were determined. Degraded samples have reduced 28 S/18 S ratios and contain additional small RNA fragments that appear as smears in the gel photo and small humps in the electropherogram making them easily detectable. Samples were discarded if the total RNA profile displayed evidence of significant degradation, as indicated by a ribosomal RNA peak ratio (28 S/18 S) below 1.0 (Applied Biosystems/Ambion TechNote 11:2). No samples were lost due to RNA degradation provided the acutely dissociated cells were sorted into collection tubes that contained RNALater™. The typical yield of total RNA was ~700 ng per 100,000 cells. Approximately 100 ng of total RNA was used for transcriptional profiling.

### Transcriptional profiling and quality assessment

The Affymetrix Gene 1.0 ST Array (Affymetrix, Santa Clara, CA) provides whole-transcript coverage of 28,853 mouse genes. Each gene is represented on the array by 27 probes that interrogate the full length of the gene, rather than 3’-biased interrogations used in prior technologies (e.g., the Murine U74v2 set). Purification of RNA, probe generation, chip hybridization and array scanning was performed at the SUNY Microarray Core facility. Amplified and terminal-labeled cDNA were generated by the WT Sense Target Labeling Protocol (Affymetrix). cDNA was hybridized to the array with the Affymetrix Fluidics Station 450, and scanned with the Affymetrix GeneChip® Scanner3000. Spike controls were used to determine the quality of the hybridization. A 3’/5’ probe signal ratio significantly greater or less than 1 was interpreted as indicating RNA degradation, in which case the experiment was repeated.

### Quantified in situ patterns (QISPs) and network analysis

QISPs were generated by measuring the optical density of in situ images derived from Genepaint.org, a public database of in situ hybridizations performed on E14.5 mouse embryos [23]. The sampled regions of interest (ROI) spanned the cerebral wall from the marginal zone (MZ) to the VZ. A single ROI was first placed in rostral cortex from an in situ section that included olfactory bulb as an orienting landmark. This original ROI was then subdivided into 20 sub-regions (sROI_1-20_), each sub-region corresponding to 5% of the cortical wall, and the optical density of each sROI was measured. The measured sROI optical density was corrected by subtracting background optical density, and each sROI was then averaged with adjacent sROIs to smooth the QISP profile (Figure 1).

### Hierarchical clustering of QISPS using Pearson’s correlation

The 20 optical density measurements corresponding to each QISP (sROI_1-20_) were imported into Multiple Experiment Viewer [27,28] and normalized for grouping. Multiple Experiment Viewer can cluster data by Euclidean distance or Pearson’s correlation. Pearson’s correlation quantifies the covariance between samples producing values from -1 to 1. As such it is well suited to quantify the relationship between two QISPs. Importantly two QISP with identical profiles across sROIs_1-20_ but different magnitudes will have a correlation value of 1 and be tightly clustered, thereby facilitating the analysis of co-regulated genes independent of the absolute expression levels of each.

### IPA analysis of gene groups

The IPA network analysis tool (Ingenuity Systems, Redwood City, CA) was used to analyze clustered genes. IPA is a literature-based analysis tool that uses relationships derived from reviewed journal articles as well as additional sources (e.g, KEGG, Gene Ontology) to generate networks of interactions within a data set. Clustered genes were imported and network relationships were ascertained using the “Direct and Indirect interactions” and included “Endogeneous Chemicals” (e.g., retinoic acid).

## Results

### General analysis of the E14.5 excitatory neuron transcriptome

Previous work from our lab indicated that although Eomes (Tbr2) protein is transiently expressed in the subventricular zone (SVZ), GFP from the transgene endures in post-mitotic neurons for several days after the downregulation of endogenous Eomes [22]. Thus the GFP + neuron population in this system includes not only IPCs from the SVZ, but also migrating and differentiating excitatory cortical neurons. FAC sorting purified this GFP + population to near homogeneity; analytic re-sorting of FAC sorted GFP + cells indicated a purity of > 99%. mRNA was successfully isolated from both the GFP + and GFP- cell populations, processed and profiled on the Affymetrix Gene 1.0 ST Array (Figure 1). To further improve the analytic stringency we restricted analysis to genes with an expression level of RMA (robust multichip average) ≥ 7 in the GFP + population. This threshold was based on an internal standard; for 10 eye-specific genes ([29]; Additional file 1) we calculated from the GFP + population a RMA value of 5.5 ± 0.7 (mean ± SD). All analyzed genes in this report are thus expressed at a minimum of 2.8 fold over this empirically-defined “not expressed” RMA level.

**Figure 2:**
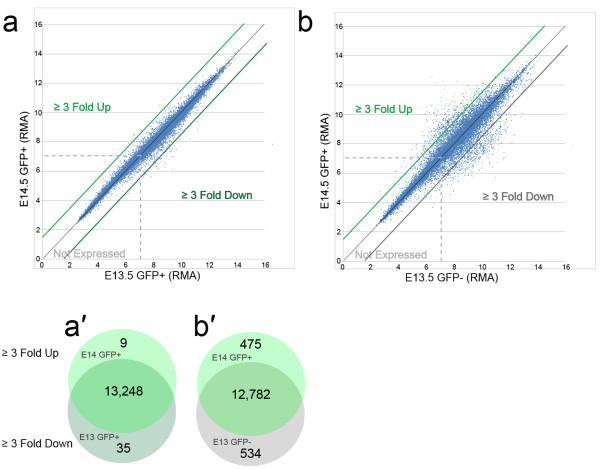
**Comparison of RMA values between samples. *a***, *a*’, Comparison of RMA values between E14.5 GFP + and E13.5 GFP + cells. ***b***, *b’*) Comparison of RMA values between E14.5 GFP + and E13.5 GFP- cells.

The total transcriptome of the E14.5 GFP + and E13.5 GFP + population was examined. We identified transcript that showed ≥ 3-fold difference (up or down) between E14.5 GFP + and E13.5 GFP + (Figure 2). The gene expression was similar at the two time points: we found 9 up-regulated and 35 down-regulated transcripts (Figure 2a, 2a’ and Additional file 2 and Additional file 3). We chose to focus our further analysis on the E14.5 GFP + data, as this is the same developmental time point used by the Genepaint mouse in situ database (http://www.genepaint.org). For the E14.5 excitatory neurons more than 13,000 unique mRNAs, out of ~25,000 represented by the 1.0 ST Array, were above the defined threshold (RMA = 7.0). Although numerous (i.e., 53% of all unique mRNAs are expressed above threshold) it is similar to recent, independent estimates of cortical neuron transcriptome size (66%, [30]; 60-70%, [31]). The difference between estimates may arise from distinct threshold assignments as well as differences in sampling technique; that is, purified FAC sorted embryonic neurons versus dissections of postnatal cortical tissue.

In an effort to focus analysis upon genes most robustly expressed by the E14.5 GFP + excitatory neuron population, we identified 475 transcripts *up*-regulated ≥ 3-fold compared to the E13.5 GFP- reference sample (Figure 2b and 2b’ and Additional file 4). An additional 534 genes were identified as being *down*-regulated by ≥ 3-fold (Additional file 5). To validate the up-regulated genes, Genepaint.org, a public database of in situ hybridizations performed on E14.5 mouse embryos, was queried [23]. Of the original 475 up-regulated transcripts, 352 were represented in the Genepaint in situ database. Fort two of the up regulated transcripts not represented by Genepaint *in situs* encoded small nucleolar RNAs, 6 encoded RIKEN cDNA clones, 5 encoded predicted genes while the remainder of non-represented genes were not readily grouped and presumably reflected Genepaint program priorities. Twenty-four of the 352 (6.8%) in situ patterns either displayed no signal in the cerebral cortex or were represented by a single in situ section with incomplete sampling of the cortical wall. The remaining 328 in situ patterns were then processed for additional validation and grouping.

#### Validation of the analytic grouping technique

A method to quantify the optical density of the in situ patterns (QISPs) across the cerebral wall was developed (see Methods). The method of QISP generation is potentially subject to error due to variability of ROI positioning and histological heterogeneity between cortical areas and sections. To assess the precision of the QISP method, multiple measurements along the rostral caudal axis were performed using a set of E14.5 in situ hybridizations for Dab1 (Additional file 6), which at E14.5 is strongly expressed in the VZ and cortical plate (CP) [32], providing two distinct regions of peak signal. The Dab1 in situ profile was measured across the cerebral wall at 5 different points within the rostral cortex on a single in situ image (Additional file 6a). The Pearson’s correlation of these five distinct quantifications ranged from 0.94 to 0.99 with an average value of 0.97 (*n* = 5). This indicates that values obtained with the ROI approach were consistent and largely insensitive to the precise position of the ROI. Similarly, we compared the Pearson’s correlation of Dab1 signal from three different, sagittal in situ images derived from sections of rostral cortex along the lateral-to-medial axis (Additional file 6b). These correlations ranged from 0.87 to 0.97 with an average correlation of 0.93 (*n* = 4). Importantly, for both the rostral-caudal and lateral-medial planes the peak signals within the VZ and CP were always restricted to within 10% of the cerebral wall (i.e., ± 2 sROI). These tests validated the QISP analysis by indicating robust concordance of optical density measurements between ROIs from different positions in the rostral cortex and along the lateral-to- medial axis.

To objectively evaluate genes of interest we quantified the in situ pattern for Eomes itself, reasoning that up-regulated genes would display a QISP equivalent to, or localized more superficially than, the QISP derived for Eomes (Tbr2). This assumption is based on the accepted observation that, at this developmental stage, the most mature neurons are located more superficially in the cortex than immature neurons [33]. Eomes displayed little expression in ventricular sROIs_1-2_ and higher expression in the more superficial sROIs_3-11_. We therefore compared the average in situ expression in sROIs_1-2_ versus the average expression in sROIs_3-20_. Transcripts where this ratio exceeded 1.0 (i.e., where expression was strongest in the deep VZ) were considered invalid. This procedure identified 11 invalid genes which were then removed from the initial, post-Genepaint list of 328 genes. Thus 97% (*n* = 317) of the ≥ 3-fold up-regulated, RMA ≥ 7 transcripts displayed a Genepaint-derived in situ hybridization pattern that was spatially consistent with the Eomes + excitatory neuron lineage.

#### Analytic grouping of the E14.5 excitatory neuron transcriptome

To explore in greater detail groups of spatially co-expressed genes, the 317 QISP-validated genes were imported into the Multiple Experiment Viewer [27,28], the signal (expression) values normalized by gene, and then hierarchically clustered. This procedure groups together similar QISPs, and can reveal groups of genes that are spatially co-expressed and thus potentially co-regulated [34].

**Figure 3:**
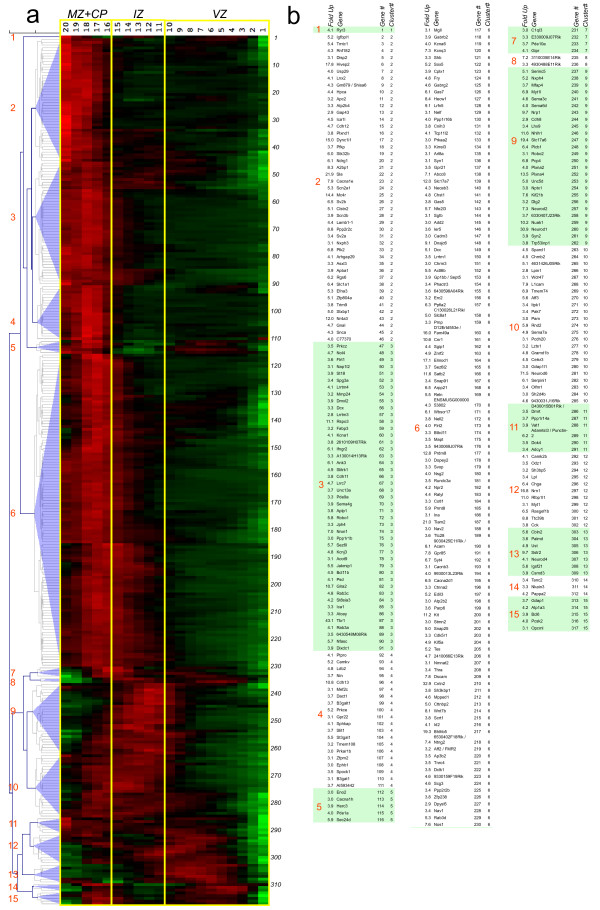
**Identification and hierarchical clustering of QISPs. *a***, Hierarchical clustering of 317 QISPs representing transcripts expressed ≥ 3 fold higher in Eomes + (GFP+) neurons, compared to GFP- cells. From left to right: gene clusters denoted numerically (1-15); expression levels in the MZ + CP, IZ, and VZ (red indicates high expression, green indicates low expression) and gene number (1-317). ***b***, Gene lists of the clustered QISPs from panel *a*. QISPs were imported into Multiple Experiment Viewer (MeV) and clustered using Pearson’s correlation (see Methods). White and shaded areas demarcate the different gene clusters.

The clustering of similar QISPs revealed several distinct spatial distributions of dynamically regulated genes in the radial dimension of the developing cerebral cortex (Figure 3). Approximately 70% of up-regulated QISPs within the Eomes + lineage display peak expression in the CP and upper intermediate zone (IZ). The majority of these QISPs displayed similar (i.e., tightly clustered) expression patterns, characterized by gradually increasing expression in the IZ and peak expression in the CP. The most heterogeneous expression patterns were observed in those QISPs with peak expression in the VZ and IZ, with a number of QISPs displaying dual expression peaks (e.g., Gdap1, Bcl6) or expression restricted to either the IZ or VZ (e.g., Neurod1 or Sstr2).

Four groups of genes – that is, four groups of clustered QISPs – were examined using a network analysis tool (IPA, Ingenuity Systems). The four groups were: (1) all 317 genes; (2) 44 “deep group” genes that achieve highest in situ expression in the VZ; (3) 49 “middle group” genes that achieve highest expression in the IZ and lower CP; and (4) 224 “superficial group” genes with greatest expression in the CP. This approach was adopted because neurons in the deep, middle, and superficial regions of the cortex have distinct morphologies and modes of migration [1] and thus can be expected to manifest dynamic regulation of genes related to morphology, navigation and migration.

#### Network analysis of all up-regulated genes

Network analysis of all 317 genes revealed 164 associated with neurological disease and 85 associated with psychological disorders; i.e., ≥ 52% of the genes have previously been implicated in human health (Table 1). One hundred thirteen of the genes were categorized as Nervous System Development and Function, indicating a substantial number (36%) of genes in this group having been previously identified and functionally characterized. The top “Molecular and Cellular Functions” (using the IPA terminology) include Cell to Cell Signaling and Interactions, Cellular Assembly and Organization, and Cellular Movement, with the top “Canonical Pathways” being Axonal Guidance Signaling and CDK5 signaling. Both of these signaling pathways are known to be active during cortical neuron migration and differentiation [35-37]. This network analysis showed that the entire ≥ 3-fold up-regulated gene category contains a large number of annotated genes associated with neuronal development, migration, differentiation, and neurological disease.

**Table 1:** Summary of IPA Ingenuity analysis for the upper, middle, and deep cortical zones

**IPA Ingenuity Pathway Analysis 317 GENES (ALL)**				
**Diseases and Disorders**	**p-value**			**#genes**
Neurological Disease	2.40E-21	-	1.99E-02	164
Psychological Disorder	3.15E-16	-	1.99E-02	85
Genetic Disorder	7.48E-16	-	1.99E-02	213
Skeletal and Muscular Disorders	3.73E-15	-	1.99E-02	123
Gastrointestinal Disorder	8.25E-15	-	1.99E-02	133
**Molecular and Cellular Signaling**	**p-value**			**#genes**
Cell-To-Cell Signaling and Interaction	7.26E-20	-	1.99E-02	68
Cellular Assembly and Organization	5.56E-16	-	1.99E-02	82
Cellular Movement	1.28E-12	-	1.99E-02	44
Molecular Transport	2.71E-07	-	1.99E-02	67
Small Molecule Biochemistry	2.71E-07	-	1.99E-02	54
**Canonical Pathways**	**p-value**			**Ratio**
Axonal Guidance Signaling	1.26E-06			23/433
CDK5 Signaling	3.87E-05			9/94
Cardiac b-adrenergic Signaling	4.55E-05			11/154
Corticotropin Releasing Hormone Signaling	2.09E-04			9/136
Dopamine Receptor Signaling	6.05E-04			7/95
**224 UPPER GENES**				
**Molecular and Cellular Signaling**	**p-value**			**#genes**
Cell-To-Cell Signaling and Interaction	4.31E-17	-	2.12E-02	51
Cellular Assembly and Organization	3.34E-11	-	2.12E-02	60
Cellular Movement	5.21E-10	-	1.42E-02	35
Cellular Function and Maintenance	4.02E-08	-	2.20E-02	49
Molecular Transport	4.02E-08	-	1.75E-02	49
**Canonical Pathways**	**p-value**			**Ratio**
Corticotropin Releasing Hormone Signaling	1.16E-04			8/136
CDK5 Signaling	1.76E-04			7/94
Synaptic Long Term Depression	1.62E-03			7/147
Cardiac b-adrenergic Signaling	2.35E-03			7/154
Axonal Guidance Signaling	2.65E-03			13/433
**49 MID GENES**				
**Molecular and Cellular Signaling**	**p-value**			**#genes**
Cellular Development	9.09E-07	-	4.47E-02	12
Cell Morphology	3.82E-06	-	3.88E-02	16
Cellular Assembly and Organization	1.16E-05	-	4.18E-02	16
Cellular Movement	4.59E-05	-	4.76E-02	9
Cell-To-Cell Signaling and Interaction	4.00E-04	-	4.76E-02	13
**Canonical Pathways**	**p-value**			**Ratio**
Axonal Guidance Signaling	1.28E-07			10/433
Semaphorin Signaling in Neurons	1.54E-05			4/52
Inositol Phosphate Metabolism	7.95E-03			3/180
CXCR4 Signaling	9.66E-03			3/169
Regulation of Actin-based Motility by Rho	2.29E-02			2/91
**44 DEEP GENES**				
**Molecular and Cellular Signaling**	**p-value**			**#genes**
Carbohydrate Metabolism	3.00E-05	-	3.88E-02	7
Molecular Transport	3.00E-05	-	3.88E-02	11
Small Molecule Biochemistry	3.00E-05	-	4.34E-02	12
DNA Replication, Recombination and Repair	1.37E-04	-	1.81E-02	2
Nucleic Acid Metabolism	1.37E-04	-	2.32E-02	7
**Canonical Pathways**	**p-value**			**Ratio**
Protein Kinase A Signaling	8.73E-05			6/328
Cardiac b-adrenergic Signaling	3.18E-04			4/154
G-Protein Coupled Receptor Signaling	1.43E-03			6/530
cAMP-mediated signaling	1.94E-03			4/219
Synaptic Long Term Potentiation	2.08E-03			3/114

In each panel the most salient “Molecular and Cellular Signaling” and “Canonical Pathways” categories are indicated, along with the associated p values. *a*, “all”. *b*, “upper group.” *c*, “middle group.” *d*, “deep group.”.

The “deep group” of 44 up-regulated genes (14% of the total), which is spatially co-expressed with Eomes (2.2 fold up), achieved highest expression in the VZ (sROIs _1-10_). This group includes clusters 1, 5, 7, 8 and 11-15 (Figure 3). Along with Eomes, the deep group is characterized by the expression of the transcriptional regulators Neurod4, Bcl6, Tanc2 and Myt1. Earlier studies focusing upon this deep area of cortex revealed (*a*) neurons with a long leading process that translocate out of the VZ [19,38,39] and (*b*) compact intermediate precursor cells in the SVZ [40]. Thus, at this point in development the GFP + cells in the VZ and SVZ are predicted to represent a mixture of intermediate precursors and postmitotic neurons [41]. Consistent with this prediction, IPA analysis identified DNA Replication, Recombination and Repair as a functional category for these group (p = 1.37E-04; Table 1, Additional file 7). Surprisingly, Carbohydrate Metabolism was also identified (p = 3.00E-05): the deep group includes genes involved in the synthesis (C1ql3 and Lpl) as well as level regulation (Chga, Gipr, Pcsk2 and Sstr2) of D-glucose. The top “Canonical Pathways” include Protein Kinase A Signaling and Cardiac β-Adrenergic Signaling. Additionally, a number of genes involved in intracellular calcium signaling (Ryr3, Camk2b, Cacna1h) showed elevated expression in this group. Network analysis of deep group genes thus suggests that the phenotypic transition from radial glial precursor to intermediate precursor/immature cortical neuron coincides with significant changes in mechanisms of glucose metabolism and second messenger signaling.

The “middle group” comprises 49 genes, or 16% of the ≥ 3 fold up data set. The middle group QISPs, comprising clusters 9 and 10, are maximal in the IZ, where developing excitatory neurons display a multipolar morphology. Multipolar neurons (MPNs) possess multiple fine processes that lack definitive orientation, and migrate relatively slowly in the radial direction [1]. The top “Molecular and Cellular Functions” in this group are Cellular Development and Cell Morphology (Table 1, Additional file 7). The middle group is dominated by the basic helix loop helix (bHLH) transcription regulators Neurod6 (71.5 fold), Neurod1 (30.9 fold), Nhlh1 (11.6 fold) and Neurod2 (7.3 fold), which represent some of the most highly up-regulated genes in the entire data set. These bHLH transcription factors have well-described functions in excitatory cortical neuron differentiation and development [6,42]. The top “Canonical Pathways” include Axonal Guidance Signaling and Semaphorin Signaling in Neurons, as well as Inositol Phosphate Metabolism. Of particular interest within the middle group genes is cluster 9, consisting of 26 genes that includes seven with known chemotropic signaling functions in axon guidance. The expression of this set of axonal guidance genes could promote the generation and extension of axons that are observed to trail the migrating cortical neuron in the upper IZ and CP [39,43]. In addition to the highly up-regulated transcription factors, the top up-regulated genes include Vglut1/Slc17A6 (19.4 fold) a glutamate transporter that identifies excitatory cortical neurons [44]. Network analysis of “middle group” genes thus indicated that developing excitatory neurons in the IZ are molecularly defined by a high expression of canonical bHLH neuronal transcription factors and axonal guidance molecules, particularly those of the Semaphorin signaling pathway.

The “upper group” of genes (*n* = 224) were observed in clusters 2, 3, 4 and 6. These genes, comprising 71% of the up-regulated gene population, achieved highest expression in the developing cortical plate (CP), an area that contains migrating neurons with an elongated or bipolar shape, and post-migratory differentiating neurons. Within the upper group the most prominent transcriptional regulators were Tbr1 (43.1 fold) [45], Bhlhb5 (19 fold) [46], and Hivep2/Schnurri2 (18 fold). Additional transcription factors in the upper group included Prdm8 [47], Satb2 [48], Sox5 [11], and Bcl11b/Ctip2 [49]. All of these transcriptions factors, with the exception of Schnurri2, have demonstrated roles in corticogenesis. Schnurri2 is, however, a BMP signaling effector, with a neuronal function suggested by the observations that mice deficient in Schnurri2 are hyperactive and exhibit hypersensitivity to stress [50].

Neurons within the forming CP migrate along radial glia fibers, from which they later detach and extend dendrites. It is therefore not surprising that the top “Molecular and Cellular Functions” in the upper group are Cell-To-Cell Signaling and Interaction, Cellular Assembly and Organization, and Cellular Movement (Table 1, Additional file 7). The top “Canonical Pathways” include Corticotropin Releasing Hormone Signaling and CDK5 Signaling. A role for CDK5 signaling mechanisms is established for many neurodevelopmental functions, including the migration of cortical neurons along radial glial fibers and subsequent cortical layer formation and dendritogenesis [51,52]. In contrast the role(s) of Corticotropin Releasing Hormone Signaling in these neurons is less clear. The eight genes from the ≥ 3 fold up-regulated category that populate the Corticotropin Releasing Hormone Signaling category include four with known functions in brain development, including the cannabinoid receptor Cnr1 [53], the transcription factor Mef2C [54], and nitric oxide synthase Nos1 [55]. The remaining genes in this group include the protein kinases Prkar1β, Prkcε and Prkcζ Network analysis of “upper group” genes thus suggest maturing excitatory neurons in the developing CP express a number of regulatory and signaling factors, including the transcription factors Tbr1, Bhlhb5, Hivep2/Schnurri2, Prdm8, Satb2, Sox5, and Bcl11b/Ctip2.

#### Analysis of highly up- and down-regulated transcription factors

**Figure 4:**
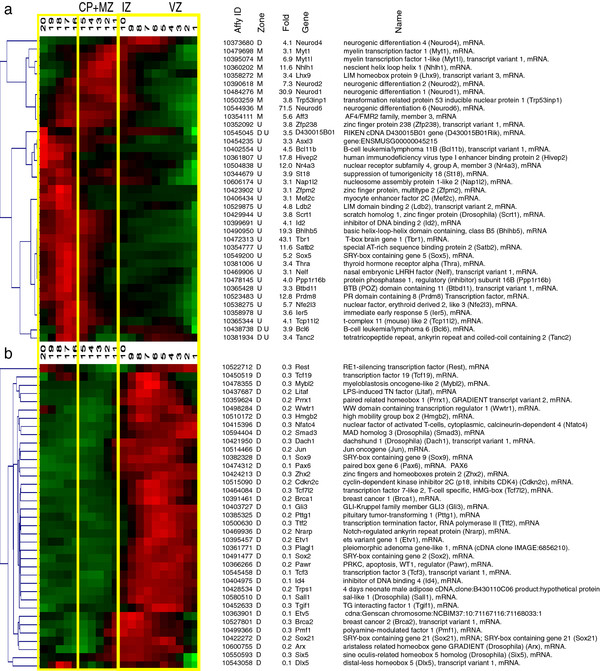
**Transcripts encoding transcription factors.** Functional groupings of QISPs based upon Gene Ontology (GO) molecular function and biological process annotations. ***a***, Clustered QISPs of transcripts expressed ≥ 3 fold higher in Eomes + neurons compared to GFP- cells. The left-most dendrogram reveals the hierarchical arrangement of QISPs. ***b***, Clustered QISPs of transcripts expressed ≥ 3 fold lower in Eomes + neurons compared to GFP- cells. *a’* and *b’.*

To explore the spatial relationships of key transcription factors (TFs) in the cortical lineage, we identified all ≥ 3 fold up- and down-regulated genes associated with the Gene Ontology descriptor “transcription, nuclear” and clustered their respective QISPs (Figure 4). These transcription/nuclear QISPs span the cerebral wall at E14.5, with subsets of QISPs showing peak expression in the VZ, IZ and CP (Figure 4a). We were initially surprised that Eomes itself was upregulated only 2.2 fold, a value below our threshold for analysis. There are several reasons for this low fold up value. The first is that GFP- cells express Eomes mRNA (RMA = 10.7). The presence of Eomes mRNA in the absence of GFP protein could imply post-transcriptional regulation of the 5’ UTR of the Eomes message that is included in the Eomes: :eGFP transgene [26]. Similarly, the presence of Eomes mRNA in GFP- cells might reflect the rapid up regulation of Eomes mRNA in a subpopulation of cells that has yet to express sufficient GFP protein for FAC detection and sorting into the GFP + group. Finally, Eomes mRNA is sharply down regulated as neurons leave the SVZ [3], while GFP protein expression persists and is expressed in all cortical zones superficial to the SVZ, at this developmental time point. Thus the GFP + cell population is likely larger than the Eomes mRNA + cell population, effectively reducing the average Eomes mRNA expression level in the GFP + population. Thus post-transcriptional regulation and/or the difference between sharp Eomes mRNA regulation and the delayed and extended GFP protein expression may account for this relatively low fold up level for this critical TF.

We first compared these genes to an earlier study of bHLH-family TFs denoted as the “Dorsal Telencephalon Network” [4]. Comparison to this independent data set of 13 TFs revealed 7 (54%) to be members of our ≥ 3 fold up-regulated category: Neurod1 (31 fold), Neurod2 (7.3 fold), Neurod6 (72 fold), Tbr1 (43 fold), Id2 (4.1 fold), Nhlh1 (12 fold) and Satb2 (12 fold) (Figure 4). Other dorsal telencephalon TFs outside the ≥ 3-fold up-regulated category include the early genes Pax6 (7.6 fold down-regulated), Neurog 1 (1.3 fold down-regulated), Neurog 2 (1.1 fold down-regulated) and Eomes itself (2.2 fold up-regulated). Although Pax6 expression in the GFP + population was below threshold (RMA = 6.2) Neurog1, Neurog2, and Eomes were not (RMA values of 7.6, 10.1, and 11.9, respectively). This suggests that the GFP + neurons in our study substantially down-regulate Pax6 relative to neuronal precursors, but do express the neuronal specifying TFs Neurog1, Neurog2 and Eomes. Additionally, two genes specific to cortical layer V-VI, Etv1 and Otx1 [56], were down-regulated in our data (5 fold and 2 fold, respectively), an observation supported by strong Genepaint in situ hybridization signals at E14.5 in the ventricular zone for both genes. Because postnatal expression of Etv1 and Otx1 is specific to cortical layer V-VI, this result suggests temporally differential (i.e., developmentally early and late) requirements for these TFs during cortical development. In contrast to the “Dorsal Telencephalon Network” we identified the layer II-IV marker Satb2 within our ≥ 3 fold up-regulated data set (12 fold), an observation confirmed by Genepaint-derived in situ hybridization. Consistent with the dorsal telencephalic identity of the Eomes + population, none of the nine genes associated with the “Ventral Telencephalon Network” [4] were observed in the ≥ 3 fold up-regulated population. By comparison to published studies of excitatory neuron differentiation [2-4,57], this TF expression analysis developmentally positions the “maturation spectrum” of the isolated E14.5 GFP + neurons to a period *after* the expression of early dorsal specification genes (e.g., Pax6), coincident with neural specification genes (e.g., Neurog1, Neurog2), and *prior* to the expression of late layer-specific markers (e.g., Etv1 and Otx1).

**Figure 5:**
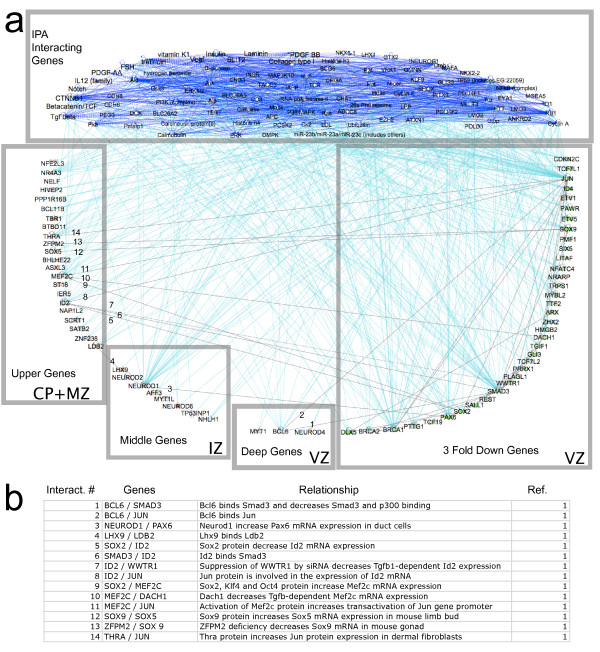
**Network analyses of dynamically regulated transcription factors (TFs) in differentiating cortical excitatory neurons, derived from the IPA-Ingenuity Network Tool (see Methods). *a***, ≥ 3 fold up- and ≥ 3 fold down-regulated TFs were imported along with endogenous chemicals (e.g., retinoic acid) to populate the network. Grey boxes collect the up-regulated genes expressed in the “upper,” “mid” and “deep” cortical regions (CP + MZ, IZ, VZ alone) as well as the down-regulated genes (VZ) and the group of IPA Interacting Genes. Gray lines that are numbered indicate interactions between cortical zones (upper, middle, and deep); light blue lines indicate direct interactions of upper, middle, and deep up-regulated genes, and down-regulated genes, with IPA interacting genes. Dark blue lines indicate interactions between IPA interacting genes (see Results). ***b***, Tabular summary of interactions between cortical zones. *1*, Interaction identified by IPA Ingenuity analysis.

We hypothesized that the highly expressed “middle” and “deep” group TFs are critical molecular regulators, perhaps functioning to simultaneously repress precursor-specific TFs and promote the expression of TFs required for proper differentiation of cortical excitatory neurons. To test this idea, a network analysis tool (IPA; see Methods) was used to explore within our dataset possible regulatory interactions among the dynamically expressed TFs (Figure 5). All TFs from the ≥ 3 fold up- and down-regulated populations were imported into the pathway analysis and the network of direct and indirect interactions was identified. All interacting genes that were identified, but absent from the populations of ≥ 3 fold up- and down-regulated TFs, were assigned to a group denoted “IPA Interacting Genes.”

Approximately 430 connections were identified for TFs in the up- and down-regulated groups.

Only 4 direct interactions were reported between the “deep group” or “middle group” genes and either the “upper group” genes or the down-regulated genes (Figure 5). Numerous direct interactions, however (50 of 54), were detected that connected “middle” and “deep” genes to the “IPA Interacting Genes” group. Included in this group are signaling molecules that function non-cell autonomously in neuronal development: Notch signaling [58], Wnt-βcatenin signaling [59], VEGF [60], TGF β [37] and retinoic acid signaling [61]. Each of these signaling pathways has been shown to regulate either the production and/or differentiation of excitatory cortical neurons. Surprisingly, only 14, or ~3%, of all detected interactions were direct between any group of dynamically regulated TF genes (*i.e*., between the 4 lower boxes in Figure 5). The results of this network analysis support models of neurogenesis that predict complex interactions between specific cell autonomous (*e.g*. transcription regulatory networks) and non-cell autonomous signaling mechanisms (*e.g*., Notch, TGFβ signaling) during the maturation of cortical excitatory neurons.

#### Analysis of highly up- and down-regulated receptor genes

**Figure 6:**
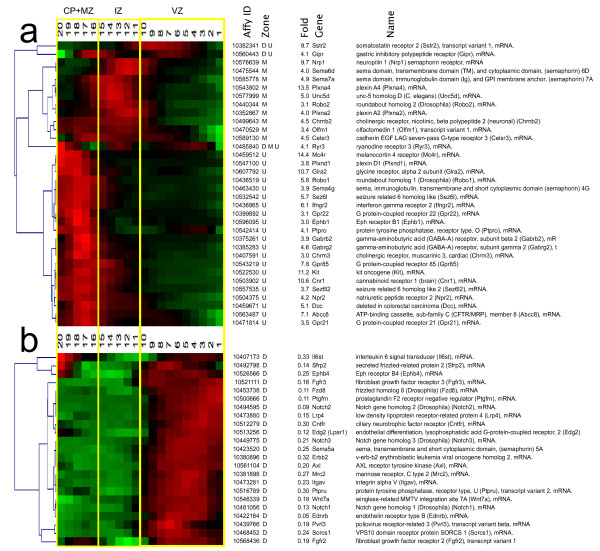
**Transcripts encoding receptors.** Functional groupings of QISPs based on Gene Ontology molecular function and biological process annotations. Graphical representation as in Figure 4. Clustered QISPs of transcripts expressed (***a***) ≥ 3 fold higher and (***b***) ≥ 3 fold lower in Eomes:: GFP + cells compared to eGFP- cells.

To more fully explore the role(s) of dynamically regulated receptors in the differentiation and migration of cortical neurons, we identified within the ≥ 3 fold up- and down-regulated gene populations those with the Gene Ontology descriptor “receptor activity,” and clustered their respective QISPs (Figure 6). The down-regulated receptors were enriched for signaling systems with established functions in neuronal precursors (Figure 6b, 6b’), including the Wnt-βcatenin signaling family members Sfrp2, Fzd8, Lrp4, and Wnt7a. Wnt-βcatenin signaling controls the production of neurons by neural precursors [62] and regulates intermediate precursor proliferation [59,63]. The FGF receptors Fgfr2 and Fgfr3, and Notch family members Notchr1, Notchr2 and Notchr3 were also down-regulated. Notch receptors have multiple roles in neurogenesis and differentiation, including the regulation of neuronal precursor differentiation [58,64], the promotion of glial fate [65], and dendritic growth [66,67]. Fgfr2 is required for excitatory neuron development [68], with Fgfr3 required for attainment of normal cortical and hippocampal volume and caudal cortex development [69]. These results suggest that, along with a dynamic enhancement of pro-differentiation genes, genes responsible for the maintenance of precursor/progenitor/glial phenotypes are dyna mically suppressed at E14.5 in the lineage of excitatory cortical neurons upon exiting the VZ.

Thirty four receptors were identified in the ≥ 3 fold up category (Figure 6a, 6a’). The top category of up-regulated receptors includes those with established roles in axonogenesis (8.14E-11 p-value): members of the Semaphorin signaling family (Nrp1, Sema6d, Plxna2, Sema7a, Plxna4, Sema4g, Plxnd1); Netrin receptors (Unc5d, Dcc); the Slit signaling receptor Robo1. Because they are observed in clusters 9 and 10 (Figure 3), many of these canonical chemotropic signaling receptors are co-expressed in the IZ. Specifically, Semaphorin signaling molecules are expressed by excitatory neurons in the IZ soon after exit from the VZ, with Netrin receptors and Robo1 expressed in more superficial domains. These chemotropic signaling systems have multiple roles and may be enabled for axonal growth [70-72], neuronal migration [73], dendritic growth [74-76] and cell survival [77].

Also included among the most highly up-regulated receptors were eight GTP-binding protein-coupled receptors (Gprs) (2.87 E-5 p-value), including Mcr4 (melanocortin receptor 4), Cnr1 (cannabinoid receptor1), Sstr2 (somatostatin receptor 2), Gipr (gastric inhibitory polypeptide receptor), and the muscarinic acetylcholine receptor Chrm3. Cnr1 has multiple developmental roles including axonal guidance [78]. Specific developmental functions of Mcr4, Gipr, and Chrm3 are unknown, although Mcr4 interacts with Cnr1 to regulate food intake in adults [79], Gipr regulates insulin secretion after feeding [80], and Chrm3 is required for pancreatic insulin and glucagon release [81] and neuronal endocannabinoid release [82]. The up-regulated Gprs also include the orphan receptor Gpr21, as well as a highly conserved receptor, Gpr85 (also known as Sreb2), known to control brain size [83]. The Flamingo homolog Celsr3, which is required for neuronal projections to the anterior commissure as well as subcerebral targets, is also up-regulated [84].

#### Analysis of highly up- and down-regulated neurotransmitter receptors and synaptic genes

Neurotransmitters and their receptors have important functions in the developing nervous system [85]. Although our dataset contained no neurotransmitter receptors down-regulated ≥ 3 fold, the up-regulated category contained a variety of neurotransmitter receptor subunits. Cholinergic receptor subunit β2 (Chrnb2) and Chrna7 are up-regulated in the IZ and CP by 4.5 fold and 2.8 fold, respectively. These receptor subunits form heteromeric receptor complexes in the developing brain, suggesting an early role for cholinergic signaling as excitatory neurons migrate out of the VZ. Although deletion of Chrna7 in mice does not cause overt structural abnormalities [86], recent studies indicate that haploinsufficiency in CHRNA7 may cause a range of neurodevelopmental phenotypes associated with the 15q13.3 syndrome, including developmental delay, mental retardation and seizure [87]. Although no glutamate receptors were upregulated ≥ 3 fold, N-methyl-D-aspartate (NMDA) receptor subunits Grina and Grin1 were mildly up-regulated (2.6 fold and 2.0 fold, respectively; data not shown). In addition to mediating adult forms of synaptic plasticity, NMDA receptors may mediate forms of spontaneous activity in the developing cortex [88] as well as structural plasticity in the late embryonic and early postnatal period [89,90]. mRNA encoding GABA (gamma-amino-butyric-acid) receptor subunits were also up-regulated, including Gabarg2 (4.6 fold) and Gabbrb2 (3.9 fold). Gabarb3 was upregulated 2.8 fold. Deficiency in Gabarg2 may be causal in Dravet syndrome, a form of childhood epilepsy [91]. GABA functions as an excitatory neurotransmitter in early development, xand deficiency in GABAA receptor subunits alter Inhibitory Post Synaptic Potentials (IPSPs) but not synapse number [92]. During early periods GABA can function as a depolarizing neurotransmitter and may relieve Mg^2+^ blockade of NMDA receptors [93,94]. One glycine receptor subunit, Glra2 (10.7 fold up-regulated) was identified in this group, although mice lacking Glra2 display no overt abnormalities of cortical structure [95].

**Figure 7:**
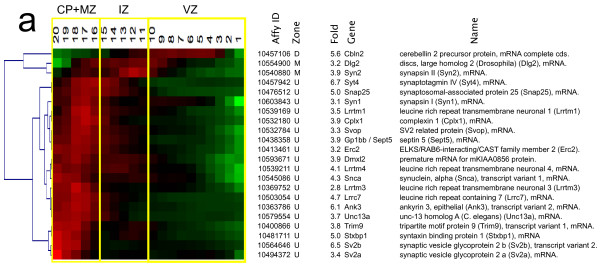
**Transcripts encoding synaptic proteins.** Functional groupings of QISPs based on Gene Ontology molecular function and biological process annotations. Graphical representation as in Figure 4. Clustered QISPs of transcripts expressed (***a***) ≥ 3 fold higher in Eomes-GFP + cells compared to GFP- cells. Note: there were no downregulated synaptic transcripts.

Synaptogenesis begins quite early in cortical development, at ~ E15 in subplate neurons in the rodent temporal cortex [96], and continues well into the postnatal period [97]. At the developmental point of our analysis, E14.5, few if any synapses are present in the developing CP. Despite this ultrastructural feature, we detected not only multiple mRNAs encoding neurotransmitter receptors, but also message for 22 other synaptic genes identified by the Gene Ontology descriptor “synapse” or “synaptic” (Figure 7). Included in this group are synaptotagmin IV (SytIV), a calcium sensing protein involved in neurotransmitter release [98], and synaptic vesicle glycoproteins Sv2a and Sv2b that may also be involved in regulated secretion [99]. Additionally detected were three members of the Leucine Rich Repeat Transmembrane family (Lrrtm1-3), which function as synaptic organizers [100]. These results admit the possibilities that neurotransmitter receptors and synaptic mRNAs expressed by these immature excitatory neurons either (*a*) do not encode functional proteins, or (*b*) encode proteins involved in non-canonical functions. In either case, the results are consistent with important roles for classical neurotransmitter signaling molecules in shaping the developmental trajectory of differentiating excitatory cortical neurons.

#### Analysis of highly up- and down-regulated voltage-dependent channels

**Figure 8:**
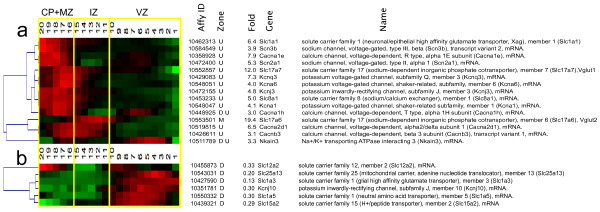
**Transcripts encoding ion channels and solute carriers.** Functional groupings of QISPs based on Gene Ontology molecular function and biological process annotations. Graphical representation as in Figure 4. Clustered QISPs of transcripts expressed (***a***) ≥ 3 fold higher and (***b***) ≥ 3 fold lower in Eomes-GFP + cells compared to GFP- cells.

Early patterns of electrical activity and calcium transients are known to shape the developing nervous system [101-104]. For this reason we queried the grouping of QISPs associated with known voltage-dependent channels (Figure 8). One down-regulated, and ten up-regulated, transcripts were identified and grouped. Of the up-regulated transcripts, four encode subunits of voltage-dependent calcium channels (Cacna1e, Cacna1h, Cacnb3, Cacna2d1), four encode voltage-dependent potassium channels (Kcna1, Kcnj3, Kcnq3, Kcna6), and two encode voltage-dependent sodium channels (Scn3b, Scn2a1). Hierarchical clustering of these transcripts’ in situ hybridization profiles revealed peak expression within the upper-most ten sROIs, corresponding to the IZ, CP and MZ. This expression pattern is spatially consistent with the acquisition of voltage-dependent ionic currents in multipolar and migrating neurons [103]. Only one gene (Kcnj10), encoding the weakly inwardly rectifying potassium channel subunit Kir4.1, was down-regulated (3.3 fold), and this observation was consistent with the corresponding in situ hybridization data. Although robustly down-regulated at this developmental time point by differentiating excitatory cortical neurons, we note that deficiency in KCNJ10 produces seizures, sensorineural deafness, ataxia, mental retardation, and electrolyte imbalance (SeSAME syndrome) [105,106]. This analysis is consistent with an important contribution of electrical activity toward shaping the development of cortical neurons (and presumably their synaptic/functional networks), but also implicates specific channel types and sub-units in the manifestation of these early activity-dependent processes by cortical excitatory neurons.

#### Analysis of highly up- and down-regulated adhesion molecules

**Figure 9:**
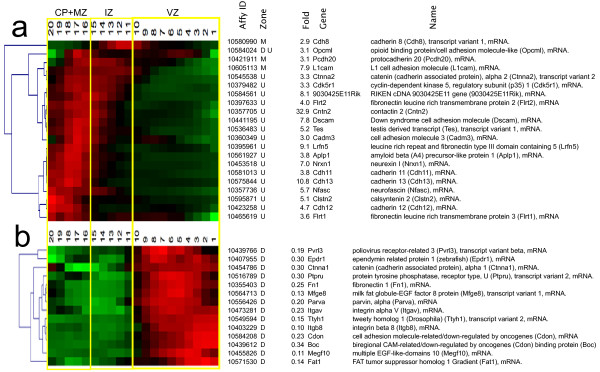
**Transcripts encoding adhesion molecules.** Functional groupings of QISPs based on Gene Ontology molecular function and biological process annotations. Graphical representation as in Figure 4. Clustered QISPs of transcripts expressed (***a***) ≥ 3 fold higher and (***b***) ≥ 3 fold lower in Eomes-GFP + cells compared to GFP- cells.

Spatial distributions of adhesion molecules, and changes in such distributions, can in principal convey substantial morphologic and migratory changes upon developing neurons. To group expression patterns of known adhesion molecules within the Eomes + neuronal lineage, we sorted within the ≥ 3 fold up-regulated category for the Gene Ontology descriptor “adhesion” (Figure 9). Twenty one genes were identified that were verified by screening the Genepaint database. These genes include several Immunoglobulin Super Family Members (IGSFMs), such as L1cam, Dscam, Nfasc, Cadm3, Cntn1 and Cntn2. All of these IGSFM genes have confirmed neurodevelopmental functions: L1cam is involved in neuronal migration and neurite outgrowth [107]; Dscam is a co-receptor for Netrin signaling and is required for commissural axon pathfinding [108]; Cadm3, also known as Nectin-like 1 or Syncam3, is a cell adhesion molecule involved in axonal myelination [109]; Nfasc and Cntn1 interact to promote the formation of paranodal axo-glial junctions during axonal myelination [110]; Cntn2, also known as Tag-1, is highly expressed on axons and can serve as a substrate for interneuron migration [111] (although Tag-1 knockout mice display no overt cortical phenotype [112], there is a disruption of paranodal myelin junctions [113]). Several Cadherins (Cadh8, Cadh11 and Cadh13) as well as Protocadherin 20 were observed to be up-regulated, although to date none of these Cadherin genes have identified roles in cortical development. Surprisingly, no members of the integrin family were observed in the ≥ 3 fold, or the ≥ 2 fold, up-regulated groups, suggesting this important family of adhesion proteins is not dynamically regulated at the transcriptional level in the Eomes + lineage at E14.5.

In contrast other adhesion molecules, such as Fibronectin (Fn1), were down-regulated in the developing Eomes + lineage. Two integrins that via heterodimerization form a fibronectin receptor (and an activating integrin for TGFβ signaling), Itgv and Itgβ8, were also down-regulated. Conditional deletion of Itgαv in radial glia and neurons causes cerebral hemorrhage [114] while reduced expression of αvβ8 integrin is associated with arteriorvenous malformations [115]. Other down-regulated genes encoding adhesion molecules were: Jam3, a tight-junction protein essential for maintaining the integrity of the cerebrovascular endothelium [116]; Cdh6, which is expressed in the VZ where it may serve to assign the cortico-striatal boundary [117]; two additional IGSFM genes, Boc and Cdon, that are evident in the VZ, where they function in Sonic Hedgehog signaling and are implicated in forms of holoprosencephaly [118,119]. Taken together these groupings suggest that expression (and suppressed expression) of numerous adhesion molecules, particularly specific members of the cadherin and IGSF families, are required elements for migration, neurite formation, and subsequent myelination of developing excitatory cortical neurons.

## Discussion

**Figure 10:**
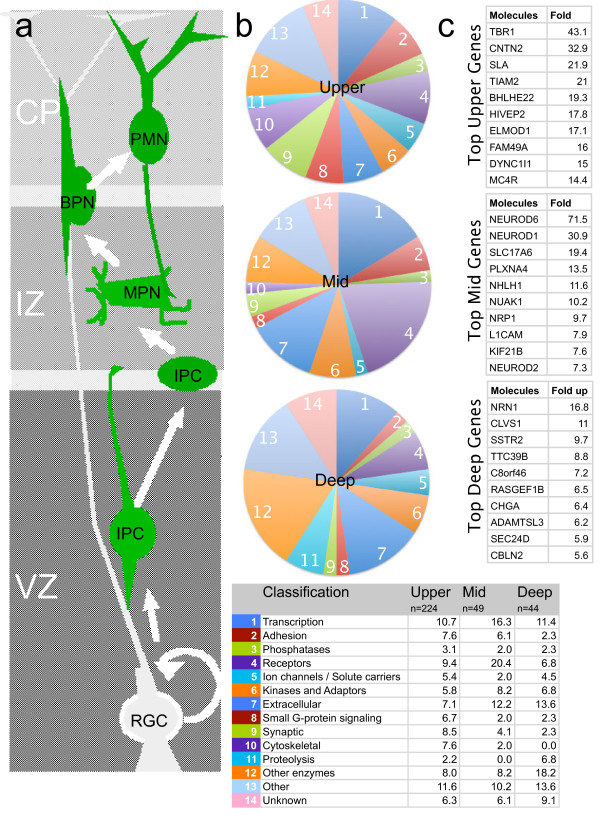
**Schematic of excitatory neuron migration, differentiation, and patterns of gene expression. *a***, Illustration of radial migration, and general morphological characteristics, of excitatory neurons. RGC, radial glial cell; IPC, intermediate precursor cell; MPN, multipolar neuron; BPN, bipolar neuron; PMN, postmigratory neuron; VZ, ventricular zone; IZ, intermediate zone; CP, cortical plate; MZ, marginal zone. ***b***, Pie charts representing gene clusters 1-14 (see Figure 3) as a function of their relative contribution to the population of ≥ 3 fold up-regulated genes as a function of cortical location (upper, middle, deep). The accompanying table (bottom) indicates the Gene Ontology (GO) classification for each of the gene clusters, and the percentage of genes within each GO classification in each zone (D, M, U). ***c***, The individual, greatest up-regulated genes in the Eomes lineage for each cortical zone.

At E14.5 the cerebral neocortex is composed primarily of neuronal precursors and immature neurons, with a small number of differentiated preplate neurons. To better identify specific patterns of gene expression within the excitatory neuron lineage during early cortical development, we combined FAC sorting and transcriptional profiling with a new, comprehensive in situ hybridization validation procedure, termed QISP. This approach revealed 317 validated genes in the ≥ 3 fold up category of transcripts. We then clustered the QISPs into groups of spatially co-expressed genes that can contribute to the combined processes of migration and differentiation. This approach allowed us to identify 44 genes expressed in the deep VZ that are likely to be IPC specific. These deep genes would be difficult to isolate by a microdissection based profiling and in situ approach [13-15] because of the mixture of cell types and maturity. Similarly, we identified a group of 49 genes that are expressed in the IZ and may contribute to the recently identified multipolar neuron stage cortical development [1], as well identifying 224 genes expressed in the developing cortical plate that in part contribute to the early morphologic differentiation of cortical excitatory neurons (Figure 10). This approach allows inferences about the temporal sequence of neuronal development: While the in situ analysis was performed at one time point (E14.5) and is formally interpreted as a spatial pattern of gene expression at that time point, an approximate developmental timeline of gene expression within the lineage can be inferred, based on the well-established observation that more superficial neurons in the developing mammalian cortex are more mature [33,120]. This inference is particularly valid at early developmental time points, such as E14.5, when the CP is starting to form and few neurons have “settled” or been displaced to deeper positions by subsequent, later-born neurons.

One of the initially surprising features of the data set is the large number of genes that exceeded our empirical expression threshold (RMA 7.0). We found that 53% of all unique mRNAs are expressed above this threshold. Although initially surprising, this result is consistent with prior studies of more mature cortical tissues [30,31] that contain a mixture of fully differentiated neurons with differing molecular properties. Although mRNA expression is not equivalent to protein expression, the fact that newly-generated excitatory cortical neurons display a complex transcriptome points to the potential plasticity of these cells from their earliest developmental origins. The broad spectrum of expressed receptors indicates chemosensitivity to major chemotropic signaling mechanisms (Semaphorin, Ephrin, Netrin, and Slit) and classical neurotransmitters including GABA, Glutamate, Acetylcholine and Glycine. Immature excitatory neurons are therefore potentially broadly sensitive to environmental cues, and could harness these cues to guide, and ultimately define, their early differentiation and migration across the cerebral wall. While the large number of signaling pathways underscores the potential plasticity and adaptability of immature neurons, this observation also highlights their potential susceptibility to toxic insult and drugs of abuse.

The Eomes::eGFP transgene causes GFP expression for a defined interval (approximately 3 days) in the early postmitotic life of excitatory neurons. Our analyses showed that the gene expressed by neurons during this ~ 3 day period of postmitotic life, is similar regardless of whether the GFP + cells were isolated from E13.5 or E14.5 embryos (Figure 2a and 2a’). This presumably reflects the shared initial developmental trajectory of these early born neurons, regardless of birthdate. This finding supports the observation of a core excitatory neuron developmental program centered on the transcription factors Pax6, Eomes and Tbr1 [57]. Thus, while our analyses focused on the comparison of E14.5 GFP + cells to GFP- precursors, we expect that many (but not all) of the genes we identified as upregulated in the transition from precursor to immature deep layer neuron will also be upregulated during the differentiation of later born, upper layer cortical neurons.

The complex interplay between the developing neuron and its environment was highlighted in our analysis of the network of transcription factors (TFs) that drive excitatory neuron differentiation. Although we were able to identify a total of 74 dynamically regulated TFs in the excitatory lineage, only 3% of the IPA literature-based interactions connected these TFs between different spatial zones (i.e., “deep,” middle,” and “upper” cortical plate). The remaining ~97% of interactions connected dynamically regulated TFs to a group of “IPA Interacting Genes,” a collection that included a number of molecules involved in non-cell autonomous signaling, such as Tgfβ, Vegf, and Wnt- βcatenin signaling systems. While this analysis only reports *known* interactions, and is therefore likely to under represent the true number of functional TF-TF interactions, it is presumably also likely to under represent indirect molecular interactions. So instead of a network composed exclusively of sequentially active (or activated) TFs, our data is consistent with an emerging model of early cortical neuron differentiation that predicts interaction between cell autonomous and non-cell autonomous mechanisms [102,121-124]. This model helps account for one of the paradoxical features of neuronal development: the stochastic cell type composition of clonally related neurons observed in, for example, the neural retina [125-127] and cortex [128,129]. In contrast to the invariant lineages that would be expected from a strictly deterministic developmental program, the variable mixture of neuronal subtypes within each clone may indicate a “noisy” deterministic program and/or a developmental program that relies on non-cell autonomous cues (e.g., growth factors or neurotransmitters) that may change in functional abundance and/or localization during development.

Another important result emerging from recent work is that specific members of TF families control restricted sets of phenotypic attributes. For example, Neurog2 enacts a program of motility in migrating neurons in the IZ through activation of the Rho family GTPase Rnd2 [130]. Later in development a second bHLH TF, Ascl1, regulates the transition to locomotory migration on radial glial fibers via Rnd3 activation [131]. Additionally, the subcortical projection patterns of cortical neurons depends on the expression of Sox5 [11] and may involve other TFs as well [7,132,133]. This stepwise control of neuronal phenotype may enable the appropriate maturation of neurons, independent of the size of the developing cerebral cortex or the time required for neurons to migrate across the cerebral wall. During early corticogenesis, mouse layer 6 neurons can transition from cell cycle exit to a postmigratory differentiating neuron in ~1.5 days [22,134], whereas layer 2/3 cortical neurons require 4-5 days for this transition [135]. Nevertheless, a similar, if not identical, TF program is thought to underlie the maturation of both classes of neurons [3,57]. Developing neurons’ interactions with external cues that promote (or suppress) the expression of stage-specific TFs would thus permit the coordinated transitions of neuronal phenotype (e.g., multipolar neuron to bipolar migrating neuron) in a manner independent of a cell-intrinsic “timer” of differentiation.

In this regard the cluster of genes expressed in the IZ may be important for coordinating the multipolar neuron stage, an early stage of migration, and later axon initiation. In clusters 9 and 10 we observed a significant enrichment of the Gene Ontology categories Cellular Movement Signaling (p = 4.59E-05) as well as Axonal Guidance Signaling Molecules (p = 1.28E-07) and bHLH TFs including Neurod6 (72 fold) and Neurod1 (31 fold). Neurod6 in particular may have a role in regulating the mitochondrial mass increase and cytoskeletal rearrangements prior to axonal growth [136], while other bHLHs, including Neurod1, promote the expression of genes critical for cortical migration, including the microtubule associated protein Dcx, the Cdk5 regulatory subunit p35, and the small GTPase RhoA [137]. The multipolar neuron phenotype could thus be explained, in part, by cellular specification provided by Neurod1 and Neurod6, two of the most highly upregulated TFs in our data set.

## Conclusion

In summary, this study provides a rigorously validated list of transcripts expressed by immature excitatory neurons that have been grouped by in situ expression pattern. We identified the transcriptional complexity of these cells, and highlighted the potential functionality of these neurons with regards to the expression of specific chemotropic receptors, neurotransmitter receptors, ion channels, adhesion molecules, and synaptic proteins. The established association of many of the validated genes with human neurological disorders revealed new molecular bases for understanding how specific genes, and functional networks of such genes, control neuronal development and contribute to pathology. Future challenges include relating identified TFs and TF networks to specific developmental events, and identifying the exogenous cues that coordinate these events.

## Abbreviations

BAC: Bacterial Artificial Chromosome; CP: Cortical Plate; eGFP: enhanced Green Fluorescent Protein; Eomes: Eomesodermin; Gsat: Gensat; IACUC: Institutional Animal Care and Use Committee; IPC: Intermediate Precursor Cell; IZ: Intermediate Zone; MPN: Multipolar Neuron; MZ: Marginal Zone; QISP: Quantified In Situ Pattern; ROI: Region of Interest; sROI: sub –Region of Interest; sVZ: sub-Ventricular Zone; Tg: Transgene; VZ: Ventricular Zone.

## Competing interests

The authors declare that they have no competing interests.

## Authors’ contributions

EO and FM designed the study. EO performed the study and co-authored the manuscript with DC and AC. All authors read and approved the final manuscript.

## Supplementary Material

Additional file 1 **Comparison of QISPs from repeated measurement of Dab1 in situ expression in rostral neocortex along (*a, b*) the rostral-caudal axis and (c, d) the lateral-medial axis.** Panels *b* and *d* plot strength of the in situ hybridization label (“intensity”) as a function of cortical position. All in situ hybridizations are derived from Genepaint. Click here for file

Additional file 2 **Table S1.** RMA values for eye-specific genes that were used to establish not-expressed threshold for this study. Click here for file

Additional file 3 **Table S2.** Complete list of all ≥ 3 fold up-regulated genes in E14.5 GFP + Eomes lineage compared to E13.5 GFP + Eomes lineage. Click here for file

Additional file 4 **Table S3.** Complete list of all ≥ 3 fold down-regulated genes in E14.5 GFP + Eomes lineage compared to E13.5 GFP + Eomes lineage. Click here for file

Additional file 5 **Table S4.** Complete list of all ≥ 3 fold up-regulated genes in the E14.5 GFP + Eomes lineage compared to E13.5 GFP- precursors. Blue cells indicate genes that were unavailable or could not be analyzed by Genepaint in situ. Click here for file

Additional file 6 **Table S5.** Complete list of all ≥ 3 fold down-regulated genes in the E14.5 GFP + Eomes lineage compared to GFP- precursors. Click here for file

Additional file 7 **Functional groupings of QISPs based on Gene Ontology (GO) molecular function and biological process annotations.** Genes are grouped according to zone of expression: deep (D, dark gray), middle (M, light gray), upper (U, white). Listed genes are ranked by fold up-regulation. Click here for file
