# Supplementary figures and images for: Hierarchical clustering of gene expression patterns in the Eomes + lineage of excitatory neurons during early neocortical development
Source: BMC Neurosci. 2012 Aug 1;13:90. doi: 10.1186/1471-2202-13-90 (PMC3583225; doi:10.1186/1471-2202-13-90)

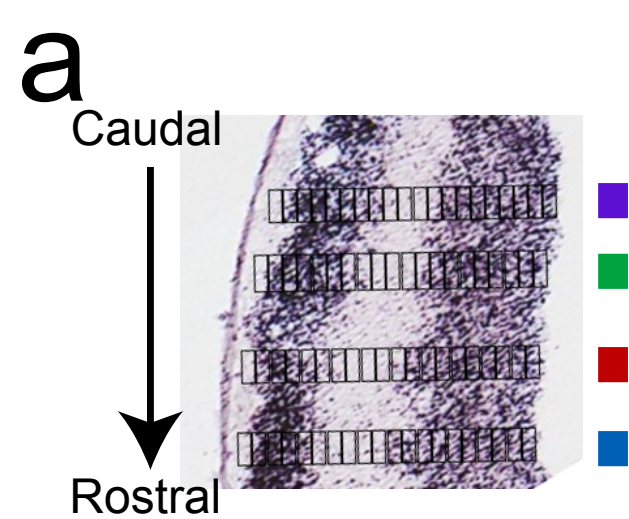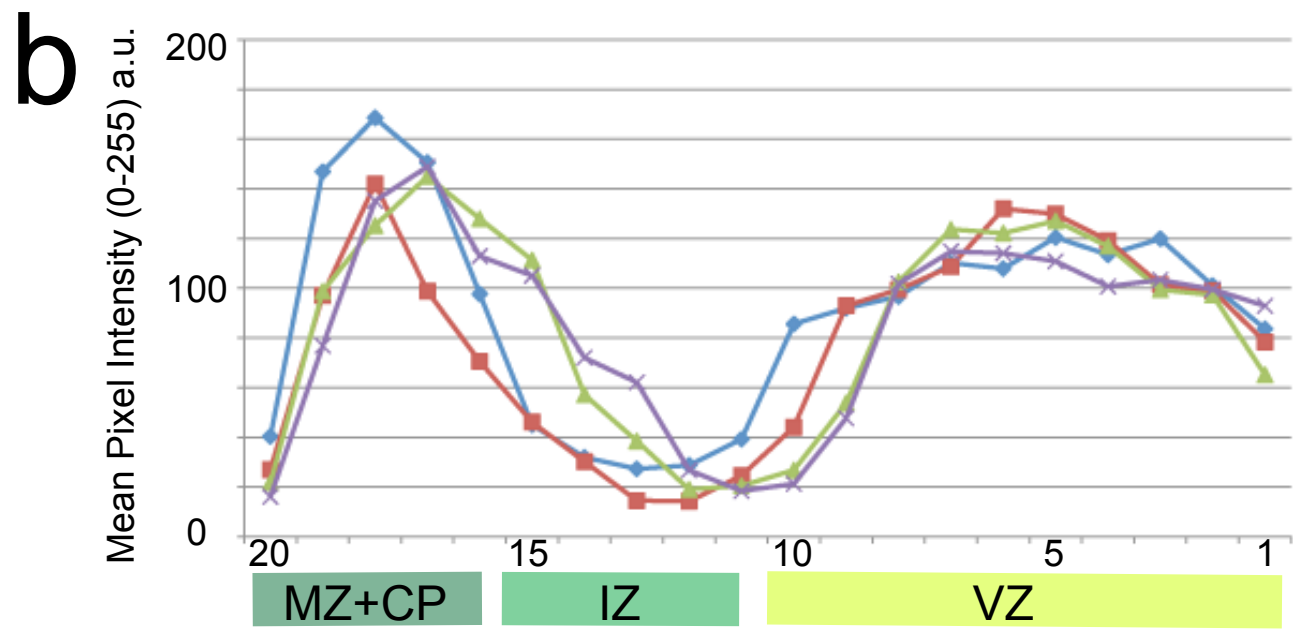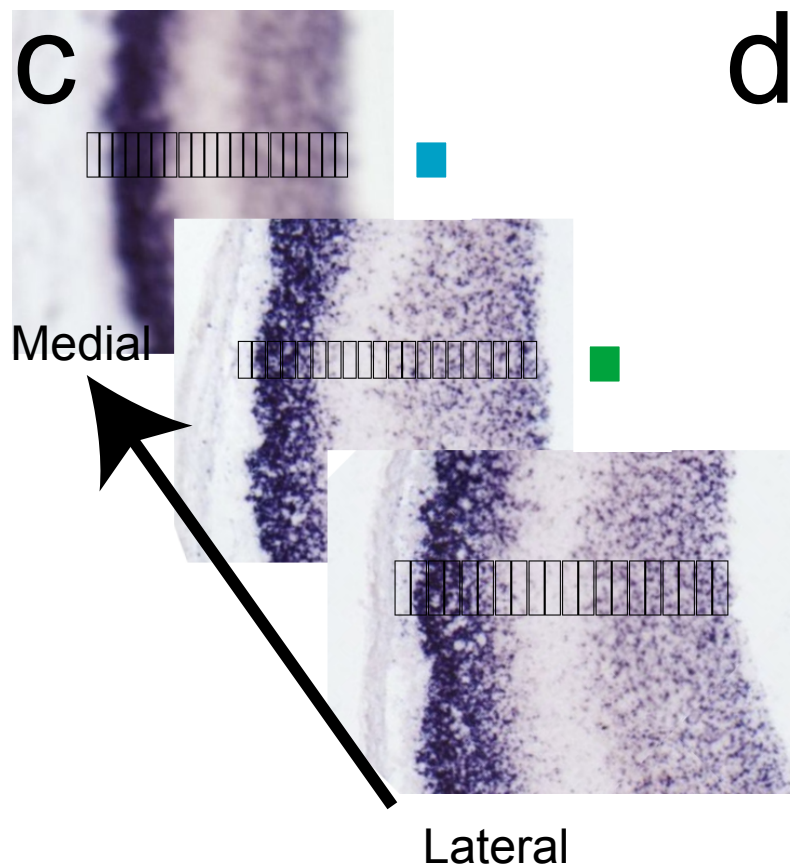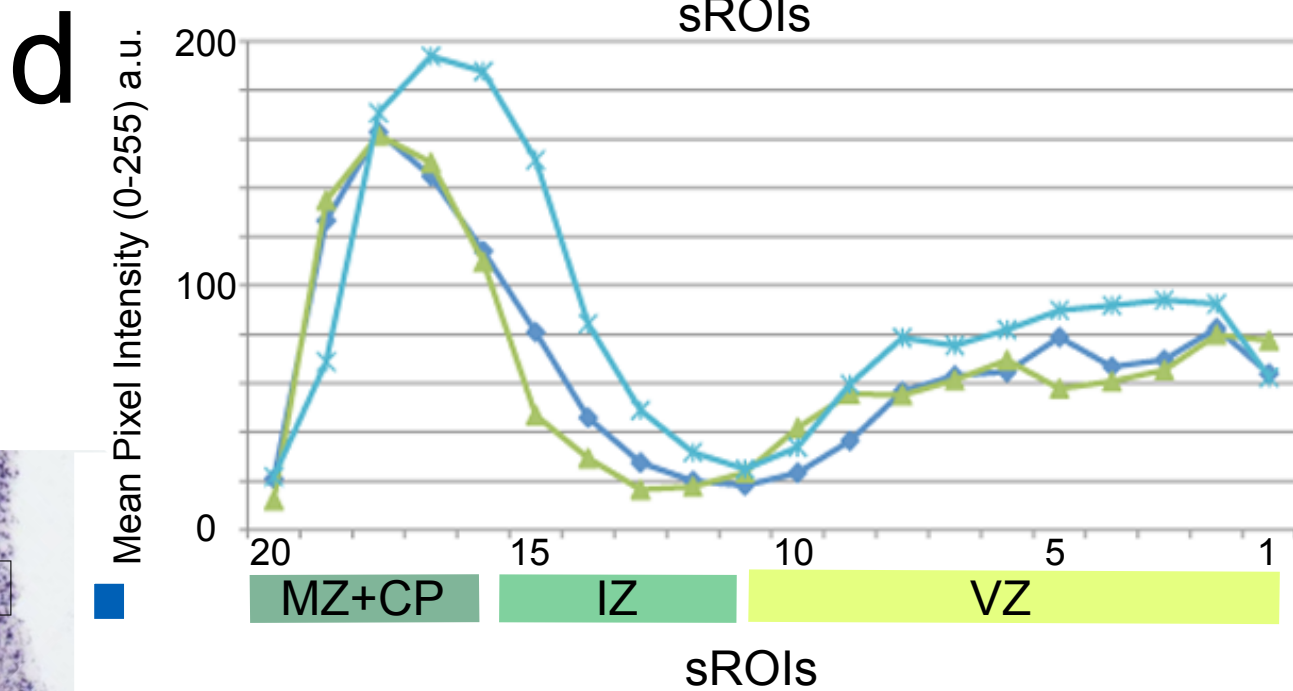

Supplement: Additional file 6 — Table S5. Complete list of all ≥ 3 fold down-regulated genes in the E14.5 GFP + Eomes lineage compared to GFP- precursors. [file 1471-2202-13-90-S6.pdf]
