# Supplementary material for: Hierarchical clustering of gene expression patterns in the Eomes + lineage of excitatory neurons during early neocortical development
Source: BMC Neurosci. 2012 Aug 1;13:90. doi: 10.1186/1471-2202-13-90 (PMC3583225; doi:10.1186/1471-2202-13-90)
Supplement: Additional file 7 — Functional groupings of QISPs based on Gene Ontology (GO) molecular function and biological process annotations. Genes are grouped according to zone of expression: deep (D, dark gray), middle (M, light gray), upper (U, white). Listed genes are ranked by fold up-regulation. [file 1471-2202-13-90-S7.pdf]

Additional File 7. Functional groupings of QISPs based on Gene Ontology (GO) molecular function and biological process annotations.
Genes are grouped according to zone of expression: deep (D, dark gray), middle (M, light gray), upper (U, white).
Listed genes are ranked by fold up-regulation.

Transcription

Fold	Gene	Zone	Gene#	Clust#
4.1	Neurod4	D	307	13
3.9	Bcl6	D U	315	15
3.5	D430015B01Rik	D U	286	11
3.4	Tanc2	D U	310	14
3.1	Myt1	D U	299	12
72	Neurod6	M	281	10
31	Neurod1	M	260	9
12	Nhlh1	M	246	9
7.3	Neurod2	M	257	9
6.9	Myt1l	M	240	9
5.6	Aff3	M	270	10
3.8	Trp53inp1	M	262	9
3.4	Lhx9	M	245	9
43	Tbr1	U	87	3
19	Bhlhb5	U	217	6
18	Hivep2	U	6	2
13	Prdm8	U	177	6
12	Nr4a3	U	43	2
12	Satb2	U	166	6
5.7	Nfe2l3	U	143	6
5.2	Sox5	U	122	6
4.8	Ldb2	U	94	4
4.5	Bcl11b	U	80	3
4.1	Id2	U	216	6
4.1	Tcp11l2	U	132	6
4	Ppp1r16b	U	130	6
3.9	St18	U	51	3
3.8	Zfp238	U	226	6
3.8	Scrt1	U	215	6
3.6	Ier5	U	146	6
3.4	Thra	U	208	6
3.3	Btbd11	U	174	6
3.3	Asxl3	U	35	2
3.1	Nap1l2	U	50	3
3.1	Zfpm2	U	107	4
3.1	Mef2c	U	97	4
3.1	Nelf	U	129	6

Adhesion

Fold	Gene	Zone	Gene#	Clust#
3.1	Opcml	D U	317	15
7.9	L1cam	M	268	10
3.1	Pcdh20	M	276	10
2.9	Cdh8	M	244	9
32.9	Cntn2	U	210	6
10.8	Cdh13	U	96	4
9.1	Lrfn5	U	128	6
8.1	9030425E11Rik	U	190	6
7.8	Dscam	U	209	6
7	Nrxn1	U	74	3
5.7	Nfasc	U	90	3
5.2	Tes	U	205	6
5.1	Clstn2	U	27	2
4.7	Cdh12	U	15	2
4	Flrt2	U	173	6
3.8	Aplp1	U	71	3
3.8	Cdh11	U	66	3
3.6	Flrt1	U	49	3
3.3	Cdk5r1	U	203	6
3.3	Ctnna2	U	196	6
3	Cadm3	U	147	6

Kinases and adaptors

Fold	Gene	Zone	Gene#	Clust#
4.1	Camk2b	D U	292	12
3.5	Dok4	D U	290	11
3.2	Sh3bp5	D U	294	12
10.2	Nuak1	M	259	9
3.4	Pak7	M	272	10
3.4	Itpk1	M	271	10
3	Sh2d4b	M	284	10
21.9	Sla	U	22	2
6.8	Plk2	U	33	2
6	Stk32b	U	19	2
5.2	Prkce	U	100	4
5.2	Camkv	U	93	4
4.1	Sphkap	U	102	4
3.8	Sh3kbp1	U	211	6
3.7	Pfkp	U	18	2
3.5	Dclk1	U	222	6
3.5	Prkcz	U	47	3
3.3	Shb	U	121	6
3	Prkar1b	U	106	4
3	Prkaa2	U	133	6

Receptors

Fold	Gene	Zone	Gene#	Clust#
9.7	Sstr2	D	306	13
4.1	Ryr3	D M U	1	1
4.1	Gipr	D U	234	7
14	Plxna4	M	252	9
9.7	Nrp1	M	243	9
5	Unc5d	M	253	9
4.9	Sema7a	M	275	10
4.5	Chrnb2	M	264	10
4.5	Celsr3	M	279	10
4	Sema6d	M	242	9
4	Plxna2	M	251	9
3.4	Olfm1	M	283	10
3.1	Robo2	M	249	9
14	Mc4r	U	25	2
11	Kit	U	200	6
11	Glra2	U	82	3
11	Cnr1	U	161	6
7.8	Gpr85	U	191	6
7.1	Abcc8	U	138	6
6.1	Ifngr2	U	62	3
5.8	Robo1	U	72	3
5.7	Sez6l	U	76	3
5.1	Dcc	U	149	6
4.6	Gabrg2	U	125	6
4.2	Npr2	U	182	6
4.1	Ptpro	U	92	4
3.9	Gabrb2	U	118	6
3.9	Sema4g	U	70	3
3.8	Plxnd1	U	16	2
3.7	Sez6l2	U	165	6
3.5	Gpr21	U	137	6
3.1	Gpr22	U	101	4
3	Chrm3	U	151	6
3	Ephb1	U	108	4

Ion channels and solute carriers

Fold	Gene	Zone	Gene#	Clust#
3.3	Nkain3	D U	311	14
3	Cacna1h	D U	113	5
19.4	Slc17a6	M	247	9
12	Slc17a7	U	139	6
7.9	Cacna1e	U	23	2
7.3	Kcnq3	U	120	6
6.5	Cacna2d1	U	195	6
6.4	Slc1a1	U	38	2
5.3	Scn2a1	U	24	2
5	Slc8a1	U	158	6
4.8	Kcnj3	U	77	3
4.1	Kcna1	U	60	3
4	Kcna6	U	119	6
3.9	Scn3b	U	28	2
3.1	Cacnb3	U	193	6

Phosphatases

Fold	Gene	Zone	Gene#	Clust#
3.7	Ppp1r14a	D U	287	11
2.8	Lpin1	M	266	10
9.1	Dnajc6	U	148	6
8.8	Ppp2r2c	U	30	2
6.5	Arpp21	U	168	6
3.4	Ppp2r2b	U	225	6
3.4	Nav1	U	228	6
3.4	Phactr3	U	154	6
3	Ppp1r1b	U	75	3

Extracellular

Fold	Gene	Zone	Gene#	Clust#
5.6	Igsf21	D	308	13
6.4	Chga	D U	296	12
6.2	Adamtsl3	D U	289	11
4	Pcsk2	D U	316	15
3.8	Cck	D U	302	12
3	C1ql3	D U	231	7
6.1	Serpini1	M	282	10
5.2	Nxph4	M	238	9
4.6	Sema3c	M	241	9
4.5	Sparcl1	M	263	10
3.7	Mfap4	M	239	9
3	Nptx1	M	254	9
11.1	Rspo3	U	58	3
8.1	Wnt7b	U	214	6
7.4	6530402F18Rik	U	218	6
5.5	Reln	U	169	6
5.3	Efna3	U	39	2
5.2	Igfbpl1	U	2	2
5.2	Edil3	U	197	6
4.6	Scg3	U	224	6
4.4	Lamb1-1	U	29	2
4.3	Necab3	U	140	6
3.8	Nell2	U	172	6
3.8	Cnih3	U	131	6
3.7	Slit1	U	103	4
3.5	Spock1	U	109	4
3.3	Kirrel3	U	134	6
3.1	Nxph3	U	32	2

Small G-protein signaling

Fold	Gene	Zone	Gene#	Clust#
6.5	Rasgef1b	D U	300	12
5.9	Rnd2	M	274	10
21	Tiam2	U	187	6
17.1	Elmod1	U	164	6
6.2	Rgs6	U	37	2
5.3	Rab3d	U	229	6
4.8	Rab3c	U	83	3
4.7	Gnal	U	44	2
4.5	Ica1l	U	14	2
4.1	Rab3a	U	88	3
4.1	Psd	U	81	3
4.1	Arhgap29	U	34	2
3.7	Nin	U	95	4
3.5	Rundc3a	U	181	6
3.4	Spg3a	U	52	3
3.3	Ica1	U	85	3
3.1	Arl8a	U	135	6

Synaptic

Fold	Gene	Zone	Gene#	Clust#
5.6	Cbln2	D	303	13
3.9	Syn2	M	261	9
3.2	Dlg2	M	256	9
6.7	Syt4	U	192	6
6.5	Sv2b	U	26	2
6.1	Ank3	U	64	3
5	Stxbp1	U	42	2
5	Snap25	U	202	6
4.7	Lrrc7	U	67	3
4.3	Snca	U	45	2
4.1	Lrrtm4	U	53	3
3.9	Cplx1	U	123	6
3.9	Dmxl2	U	55	3
3.9	Gp1bb	U	153	6
3.8	Trim9	U	41	2
3.7	Unc13a	U	68	3
3.5	Lrrtm1	U	150	6
3.4	Sv2a	U	31	2
3.3	Svop	U	179	6
3.2	Erc2	U	156	6
3.1	Syn1	U	136	6
2.8	Lrrtm3	U	57	3

Cytoskeletal

Fold	Gene	Zone	Gene#	Clust#
7.6	Kif21b	M	255	9
15	Dync1i1	U	17	2
6.1	Gas7	U	126	6
5.5	Jakmip1	U	79	3
5	Cttnbp2	U	213	6
4.9	Kif5a	U	204	6
4.9	Slitrk1	U	65	3
3.9	Dixdc1	U	91	3
3.5	Mapt	U	175	6
3.3	Dcx	U	56	3
3.3	Atcay	U	86	3
3.3	Cotl1	U	184	6
3.2	Apc2	U	11	2
3	Stmn2	U	201	6
3	Add2	U	145	6
3	Nav2	U	188	6
2.9	Gap43	U	13	2
2.9	Dpysl5	U	227	6

Proteolysis

Fold	Gene	Zone	Gene#	Clust#
4.2	Pappa2	D U	312	14
3.9	Herc3	D U	114	5
3.5	Odz1	D U	293	12
8.4	Hecw1	U	127	6
4.9	Znrf2	U	163	6
4.3	Rnf182	U	4	2
4	Usp29	U	7	2
3.7	Dact1	U	98	4

Other enzymes

Fold	Gene	Zone	Gene#	Clust#
4.9	Ust	D	305	13
3.9	Csmd3	D	309	13
4.2	Atp1a3	D U	314	15
4	Pde1a	D U	115	5
3.7	Pde10a	D U	233	7
3.4	Lpl	D U	295	12
3.4	Adcy1	D U	291	11
3	Eno2	D U	112	5
6.4	Plcb1	M	248	9
5.1	4631426J05Rik	M	265	10
4.8	Gramd1b	M	278	10
3	Pam	M	273	10
6.1	Wbscr17	U	171	6
5.9	Prmt8	U	185	6
5.5	St3gal1	U	104	4
4.8	Chst1	U	141	6
4.6	Mpped1	U	212	6
4.4	Sgip1	U	162	6
4.2	St8sia3	U	84	3
4	Nsg2	U	180	6
3.7	B3galt1	U	99	4
3.6	Parp6	U	199	6
3.3	Pde9a	U	69	3
3.2	Mmp24	U	54	3
3.2	Tmem108	U	105	4
3.1	Mgll	U	117	6
3.1	Acot9	U	78	3
3.1	Nmnat2	U	207	6
3.1	B3gat1	U	110	4
3	Atp2b2	U	198	6

Other

Fold	Gene	Zone	Gene#	Clust#
3.6	Palmd	D	304	13
17	Nrn1	D U	297	12
11	Rlbp1l1	D U	298	12
5.9	Sec24d	D U	116	5
3.9	Vat1	D U	288	11
3.7	Gdap1	D U	313	15
8.9	Tmem74	M	269	10
6.8	Pcp4	M	250	9
5.1	Serinc5	M	237	9
3.2	Lzts1	M	277	10
3	Gdap1l1	M	280	10
8.3	A2bp1	U	21	2
7.6	Nos1	U	230	6
6.3	Ppfia2	U	157	6
6.1	Ndrg1	U	20	2
5.5	Actl6b	U	152	6
4.8	Fry	U	124	6
4.7	Nol4	U	48	3
4.4	Hpca	U	10	2
4.4	Ralyl	U	183	6
4.3	Gm879	U	9	2
4.1	Lnx2	U	8	2
4	C77370	U	46	2
3.9	Apba1	U	36	2
3.8	Gas5	U	142	6
3.5	Ap3b2	U	220	6
3.5	Tnrc4	U	221	6
3.4	Snap91	U	167	6
3.3	Atp2b4	U	12	2
3.3	Prnp	U	159	6
3.3	Jph4	U	73	3
3.2	Fabp3	U	59	3
3.2	Aff2 / FMR2	U	219	6
3.1	Ina	U	186	6
3.1	Sgtb	U	144	6
3.1	Disp2	U	5	2
3	Dopey2	U	178	6

Unknown

Fold	Gene	Zone	Gene#	Clust#
7.2	3110035E14Rik	D M	235	8
3.3	4930488E11Rik	D M	236	8
8.8	Ttc39b	D U	301	12
3.3	E330009J07Rik	D U	232	7
4.6	9430031J16Rik	M	285	10
3.7	6330407J23Rik	M	258	9
3.1	Wdr47	M	267	10
16	D12Ertd553e	U	160	6
5.4	Tmtc1	U	3	2
5.1	Zfp804a	U	40	2
4.7	2410066E13Rik	U	206	6
4.6	9330159F19Rik	U	223	6
4.3	ENSMUSG00000053802	U	170	6
4	9930013L23Rik	U	194	6
3.8	2610109H07Rik	U	61	3
3.7	AI593442	U	111	4
3.6	Ttc28	U	189	6
3.6	6430598A04Rik	U	155	6
3.5	6430548M08Rik	U	89	3
3.5	9430069J07Rik	U	176	6
3.3	A130014H13Rik	U	63	3
